# Sensitivity of Human Induced Pluripotent Stem Cells and Thereof Differentiated Kidney Proximal Tubular Cells towards Selected Nephrotoxins

**DOI:** 10.3390/ijms25010081

**Published:** 2023-12-20

**Authors:** Isaac Musong Mboni-Johnston, Nazih Mohamed Zakari Kouidrat, Cornelia Hirsch, Andreas Georg Weber, Alexander Meißner, James Adjaye, Nicole Schupp

**Affiliations:** 1 Institute of Toxicology, Medical Faculty and University Hospital, University of Düsseldorf, 40225 Düsseldorf, Germany; mbonijoh@hhu.de (I.M.M.-J.); nazih.kouidrat@hhu.de (N.M.Z.K.); hirsch.cornelia@gmail.com (C.H.); alexander.meissner@hhu.de (A.M.); 2 Institute for Stem Cell Research and Regenerative Medicine, Medical Faculty and University Hospital, University of Düsseldorf, 40225 Düsseldorf, Germany; james.adjaye@med.uni-duesseldorf.de; 3 Zayed Centre for Research into Rare Diseases in Children (ZCR), EGA Institute for Women’s Health, University College London (UCL), 20 Guilford Street, London WC1N 1DZ, UK

**Keywords:** hiPSC, differentiation, regeneration, cisplatin, cyclosporin A

## Abstract

Proximal tubular epithelial cells (PTEC) are constantly exposed to potentially toxic metabolites and xenobiotics. The regenerative potential of the kidney enables the replacement of damaged cells either via the differentiation of stem cells or the re-acquisition of proliferative properties of the PTEC. Nevertheless, it is known that renal function declines, suggesting that the deteriorated cells are not replaced by fully functional cells. To understand the possible causes of this loss of kidney cell function, it is crucial to understand the role of toxins during the regeneration process. Therefore, we investigated the sensitivity and function of human induced pluripotent stem cells (hiPSC), hiPSC differentiating, and hiPSC differentiated into proximal tubular epithelial-like cells (PTELC) to known nephrotoxins. hiPSC were differentiated into PTELC, which exhibited similar morphology to PTEC, expressed prototypical PTEC markers, and were able to undergo albumin endocytosis. When treated with two nephrotoxins, hiPSC and differentiating hiPSC were more sensitive to cisplatin than differentiated PTELC, whereas all stages were equally sensitive to cyclosporin A. Both toxins also had an inhibitory effect on albumin uptake. Our results suggest a high sensitivity of differentiating cells towards toxins, which could have an unfavorable effect on regenerative processes. To study this, our model of hiPSC differentiating into PTELC appears suitable.

## 1. Introduction

The kidney is a detoxifying organ that filters and transports not only metabolic waste products from the body but also drugs and potentially toxic xenobiotics. More than 32% of the 200 most important drugs are excreted via the kidney. The epithelial cells of the proximal convoluted tubule are the major players in the reabsorption and secretion of chemical substances [1,2]. This critical role makes proximal epithelial cells (PTEC) particularly vulnerable to damage caused by xenobiotics, which can lead to nephrotoxicity. In fact, an estimated 20% of acute kidney injury (AKI) cases in hospitals are attributed to PTEC-mediated drug nephrotoxicity, making PTEC the most studied cell type in drug safety evaluations, regeneration therapies, and tissue modeling [3].

The tubular system has regeneration potential, which occurs even in the absence of specific insults to continuously replace aged cells to preserve the organ’s structural and functional integrity. However, in the event of nephrotoxicity or other damage to the kidney, the basic cell turnover is significantly exceeded by the regeneration process to repair the damaged areas [4]. This tubule cell regeneration occurs either by local stem cell proliferation or following the epithelial–mesenchymal transition (EMT) of tubule epithelial cells near the injury site [5]. Ultimately, one or a combination of these pathways will restore organ structure and function. Despite the replacement of tubule epithelial cells in the kidney, there is unambiguous evidence that the kidney does not regain one hundred percent of its former function, especially in patients with post-AKI. They have an increasing risk of developing chronic kidney diseases (CKD), suggesting that the functioning of existing tubular cells may have been compromised or those cells replacing injured cells are not fully functional upon regeneration [6,7]. Moreover, aging, oxidative stress, and toxic agents can interfere with repairing damaged areas, possibly resulting in dysfunction, and ultimately increasing the risk of renal failure [6].

Therefore, for meaningful risk assessments, it will be necessary to investigate the influence nephrotoxic substances have on renal regeneration. A potential avenue could be the development of human induced pluripotent stem cell (hiPSC)-based approaches that might serve as a model for toxicity effects during regeneration [8]. hiPSC are primarily derived from reprogrammed human somatic cells such as dermal fibroblasts. They have proven to be a robust and reproducible source of human cell types for regeneration and disease modelling, as hiPSC are capable of extensive self-renewal and can differentiate into multiple somatic cell types within the three germ layers [9]. hiPSC allow studies without ethical concerns compared to animal and human embryonic stem cell (hESC) models [9]. In addition, their use is consistent with the 3Rs principles for next-generation toxicity assessment: replacement, reduction, and refinement of animal-based experiments with new methodological approaches [8,10]. Interestingly, models using hiPSC have the potential to be more accurate in tailored toxicity assessment than traditional animal models, which have low predictive values and suffer from several limitations, including interspecies differences in transporter expression [11,12]. They could also be more suitable than hESC-derived human proximal tubule-like cells, which have a low sensitivity [1,13]. Moreover, the use of hiPSC would allow the generation of patient-specific immunocompatible tissues through personalized in vitro models [14].

Although various methods for direct differentiation of hESC and hiPSC into proximal tubular epithelial-like cells (PTELC) have been reported, the focus has mostly been on using these stem cell-derived renal cells to develop a suitable and reliable model for predicting nephrotoxicity [13,15,16,17,18]. To our knowledge, our study is the first to investigate the effects of distinct toxins on the differentiation process of hiPSC leading to tubular cells. Therefore, in this study, we adapted a previously published differentiation protocol [13] to generate PTELC and evaluated the progression of hiPSC to PTELC based on mRNA and protein expression. With this differentiation protocol, hiPSC, hiPSC differentiating into PTELC, and hiPSC differentiated into PTELC were tested for their sensitivity to selected nephrotoxins. This study confirms the potential of hiPSC to differentiate into PTELC. In the future, after ensuring that PTELC are sufficiently similar to tubular cells in vivo, experiments with hiPSC differentiating into tubular cells may provide crucial information on the harmful effects of substances on the differentiation processes required for renal regeneration.

## 2. Results

### 2.1. Differentiation of hiPSC into Proximal Tubular Epithelial-like Cells

**Figure 1:**
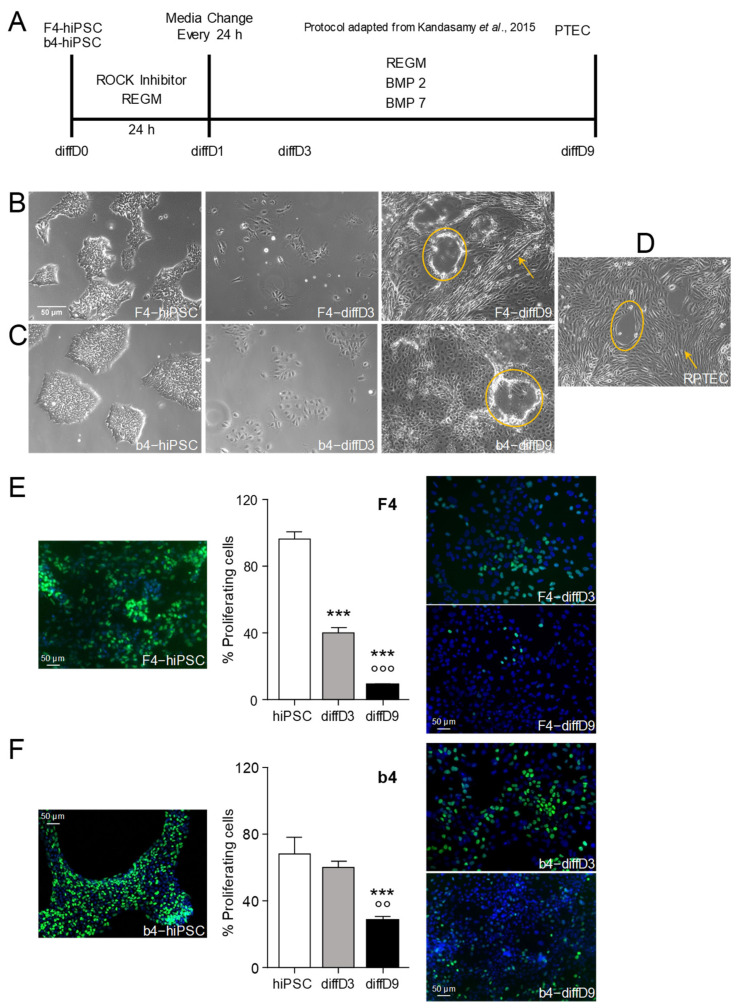
Differentiation protocol and physiological changes provoked by it. (**A**) Scheme of the treatment of the hiPSC F4- and b4 adapted from the protocol of Kandasamy et al. [13]. Morphological appearance of the F4-hiPSC and cells on day 3 and day 9 (**B**) as well as of b4-hiPSC and cells on day 3 and day 9 (**C**) of the differentiation process compared to a (**D**) commercially available kidney biopsy-derived proximal tubular cell line (RPTEC/TERT1). Reduced proliferation after initiation of the differentiation process in F4- (**E**) and b4-hiPSC (**F**), quantified by the incorporation of fluorescent 5-ethynyl-2′-deoxyuridine (EdU) into S-phase cells. The mean % of proliferating cells of 3 independent experiments and representative pictures are shown. *** *p* < 0.001 vs. hiPSC, °° *p* < 0.01, °°° *p* < 0.001 vs. diffD3 (One-way ANOVA). The scale bars represent 50 µm. Yellow circles highlight dome-like and yellow arrows show tubule-like patterns in the cell layers of the differentiated cells. BMP = bone morphogenetic protein, diffD = differentiation day, hiPSC = human induced pluripotent stem cells, PTELC = proximal tubular epithelial-like cells, REGM = renal epithelial cell growth medium, ROCK = rho-associated, coiled-coil-containing protein kinase.

Dermal fibroblast-derived hiPSC F4 and b4 were differentiated into PTELC as described in the Material and Methods with the protocol shown in Figure 1A. Both hiPSC lines underwent morphological changes from the typical cobblestone-like appearance to larger spindle-like cells within nine days (Figure 1B,C). They also formed domes and organized themselves in tubule-like patterns, features that were also observed in the human proximal tubular cell line RPTEC/TERT1 (Figure 1D).

The proliferation frequency, as monitored by EdU incorporation, was at nearly 100% in the F4-hiPSC. It showed a significant decline on differentiation day three and was down to around 10% on differentiation day nine (Figure 1E), as expected in differentiated cells. b4-hiPSC did not show as high a proportion of dividing cells as the F4-hiPSC (Figure 1F). Moreover, during differentiation, this proportion did not decrease as much in proliferation as in the differentiating F4, showing only a significant difference on differentiation day 9 (diffD9). However, the proportion of proliferating cells here was still around 30%.

**Figure 2:**
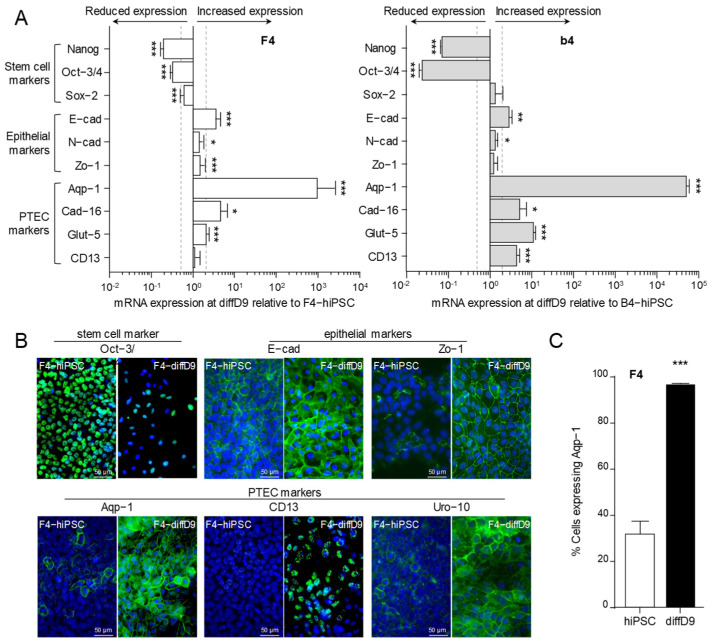
Changes in expression of differentiation markers in F4- and b4-hiPSC differentiated into proximal tubular epithelial cell-like cells (PTELC). (**A**) mRNA expression of stem cell, epithelial cell, and PTEC markers in differentiated F4- and b4hiPSC relative to expression in undifferentiated hiPSC analyzed by quantitative RT-PCR. (**B**) Visualization of selected proteins by immunocytochemical staining on F4-hiPSC and F4 on differentiation day 9. Antibodies against the different markers are visualized with FITC-coupled secondary antibodies, and nuclei are stained with DAPI. (**C**) Flow cytometry analysis of the PTEC typical protein aquaporin-1 in F4-hiPSC and F4 on differentiation day 9. Data from at least 3 independent experiments (qPCR also included 3 technical replicates) are shown as mean + SD. * *p* ≤ 0.05, ** *p* < 0.01 and *** *p* < 0.001 vs. hiPSC (Student’s *t*-test). Aqp-1 = aquaporin-1, Cad-16 = cadherin 16, CD13 = alanyl aminopeptidase, DAPI = 4′,6-diamidino-2-phenylindole, diffD = differentiation day, E-cad = E-cadherin, FITC = fluorescein isothiocyanate, Gapdh = glyceraldehyde 3-phosphate dehydrogenase, Glut-5 = fructose transporter 5, hiPSC = human induced pluripotent cells, Nanog = homeobox protein, N-cad = N-cadherin, Oct-3/4 = octamer-binding transcription factor 3/4, PTEC = proximal tubular epithelial cells, Sox-2 = sex determining region Y-box 2, Uro-10 = urothelial glycoprotein, Zo-1 = zonula occludens-1.

Expression analysis revealed a significant downregulation of mRNA expression of stem cell marker genes NANOG and Oct-3/4 after nine days of differentiation of F4- and b4-hiPSC, while Sox-2 was only significantly reduced in F4-diffD9 (Figure 2A). The expression of markers of epithelial cells, especially E-cadherin, increased in the differentiated cells compared to both hiPSC strains. Out of four analyzed markers of proximal tubular epithelial cells (PTEC), all were significantly increased in the differentiated b4. In the differentiated F4, only three of them, *aquaporin-1* (*Aqp-1*), *cadherin-16* (*Cad-16*), and the glucose transporter *Glut-5*, were significantly expressed, while *CD13* mRNA levels showed no difference after differentiation (Figure 2A). Basal expression and the expression change by differentiation of some markers were confirmed on the protein level, as seen in the immunocytochemical pictures in Figure 2B (F4) and Figure S1 (b4). This method also detected a higher increase in protein levels where little or no increase in mRNA levels in the F4-PTELC was measured, such as for CD13 or Zo-1. FACS analysis revealed that at nine days post differentiation, almost all F4-diffD9 cells expressed the PTEC marker Aquaporin-1, more than three times as many cells as compared to undifferentiated hiPSC (Figure 2C).

### 2.2. Transporter Expression and Transport Capacity of PTELC Differentiated out of hiPSC

**Figure 3:**
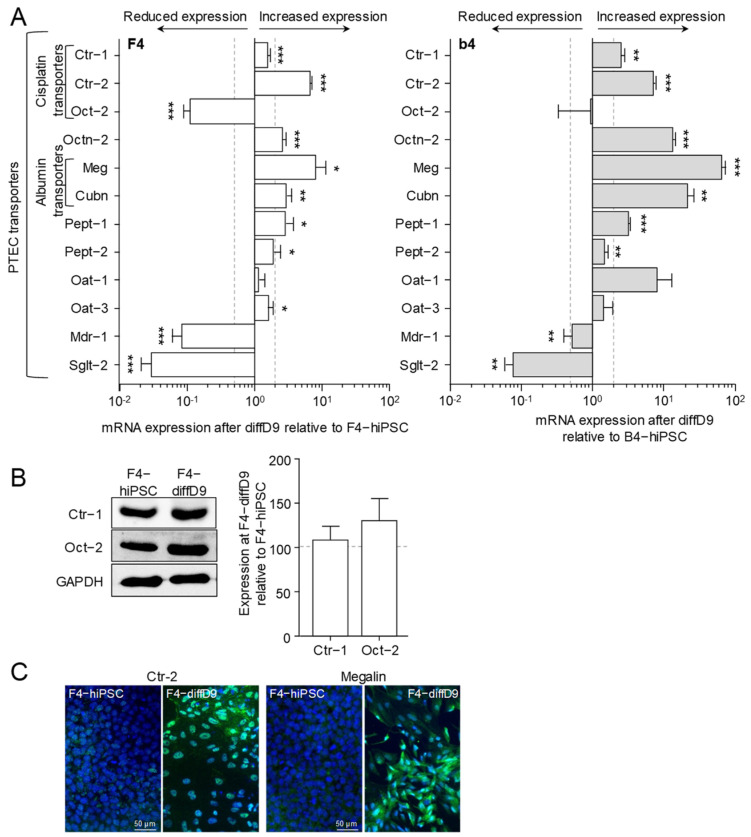
Expression changes in functional proteins in hiPSC differentiated into proximal tubule epithelial cell (PTEC)-like cells. (**A**) mRNA expression of PTEC transporters and transporters for cisplatin relative to expression in F4- (left) and b4- (right) hiPSC analyzed by quantitative RT-PCR. (**B**) Expression of selected transporter proteins in F4 cells analyzed by Western blotting. Shown are representative blots as well as their quantification. (**C**) Visualization of selected proteins by immunocytochemical staining on F4-hiPSC and F4 on differentiation day 9. Antibodies against the different markers are visualized with FITC-coupled secondary antibodies, and nuclei are stained with DAPI. Data from at least 3 independent experiments (qPCR also included 3 technical replicates) are shown as mean ± SD. * *p* ≤ 0.05, ** *p* < 0.01, *** *p* < 0.001 vs. hiPSC (Student’s *t*-test). Ctr-1/2 = copper transpor½ 1/2, Cubn = cubilin, DAPI = 4′,6-diamidino-2-phenylindole, diffD9 = differentiation day 9, FITC = fluorescein isothiocyanate, hiPSC = human induced pluripotent cells, Mdr-1 = multidrug resistance protein 1, Meg = megalin, Oat-1/3 = organic anion transporter 1, Oct-2 = organic cation transporter 2, Octn-2 = organic cation/carnitine transporter 2, Pept-1/2 = peptide transpor½ 1/2, Sglt-2 = sodium/glucose cotransporter 2.

**Figure 4:**
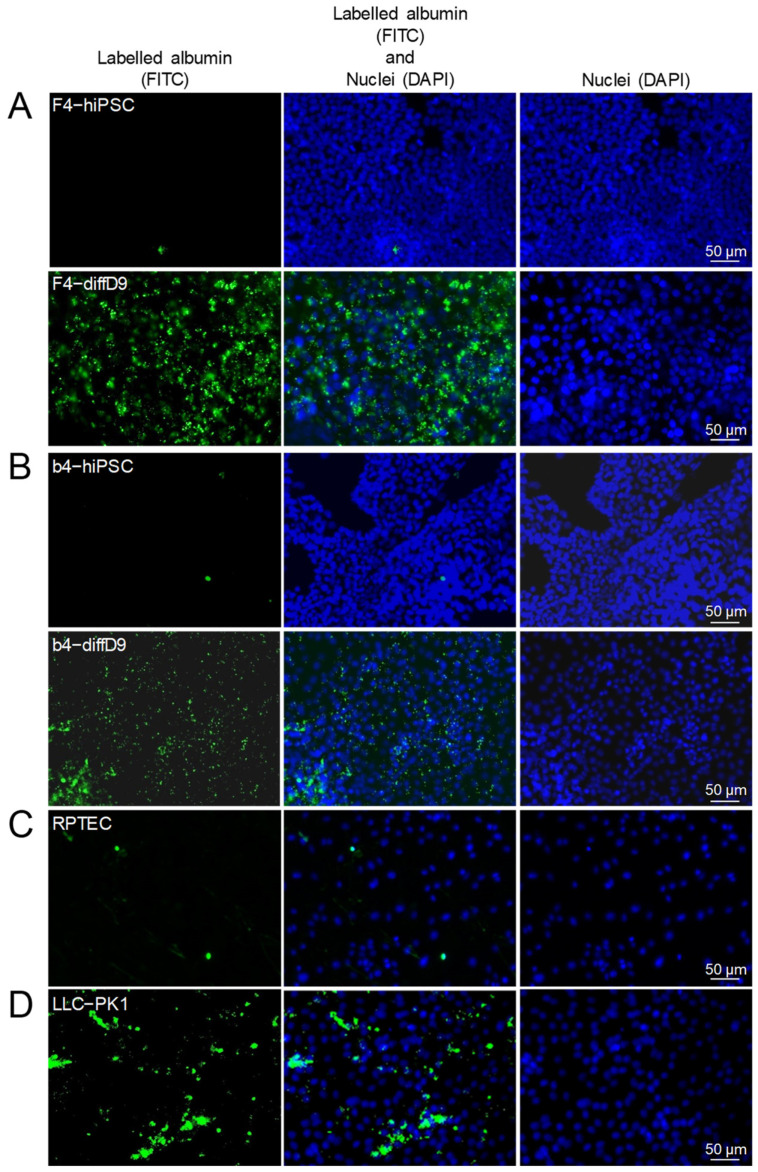
Gain of functional albumin transport in hiPSC differentiated into proximal tubule epithelial cell (PTEC)-like cells. Analysis of albumin uptake into the undifferentiated and differentiated F4- (**A**) and b4- (**B**) cells, as well as in RPTEC- (**C**) and LLC-PK1 (**D**) cells with the help of FITC-labelled BSA. Shown are representative pictures. BSA = bovine serum albumin, DAPI = 4′,6-diamidino-2-phenylindole, diffD9 = differentiation day 9, FITC = fluorescein isothiocyanate, hiPSC = human induced pluripotent cells, RPTEC = renal proximal tubule epithelial cells.

As a pre-requisite for the cells to respond to toxic substances, they must have suitable transporters to import them. Figure 3A shows that after nine days of differentiation, many of the transporters studied in F4- and b4-diffD9 were significantly more expressed than in the hiPSC, and these include transporters for cisplatin and albumin. Other transporters, such as *organic cation transporter 2* (*Oct-2*) and *multidrug resistance protein 1* (*Mdr-1*) in both F4- and b4-diffD9, were significantly downregulated. But Western blot and immunocytochemical staining confirmed the expression of the *Oct-2*, the two copper transporters *Ctr-1* and *-2*, which are crucial for the uptake of cisplatin into cells, and *megalin*, which is important for the transport of albumin, also on protein level in F4-diffD9 (Figure 3B,C). Additionally, the Western blot showed that the amount of Oct-2 was not reduced in the F4-diffD9 compared to the undifferentiated hiPSC, as quantified by PCR. F4- and b4-hiPSC differentiated for nine days into PTELC were analyzed for their ability to take up albumin as an indication of their functionality in comparison to the LLC-PK1 (porcine proximal tubular cell line, positive control) and to the RPTEC cell line (negative control). Both F4- and b4-diffD9 showed a clear uptake of labelled albumin, as depicted in Figure 4A,B, proving the functionality of the transporters for albumin. LLC-PK1 cells, on the other hand, also showed an increase in the uptake of albumin, whereas RPTEC cells did not show any significant uptake of albumin (Figure 4C,D).

### 2.3. Comparison of Marker and Transporter Expression as Well as Transport Capacity of the PTELC with the Human Proximal Tubular Cell Line RPTEC/TERT1

**Figure 5:**
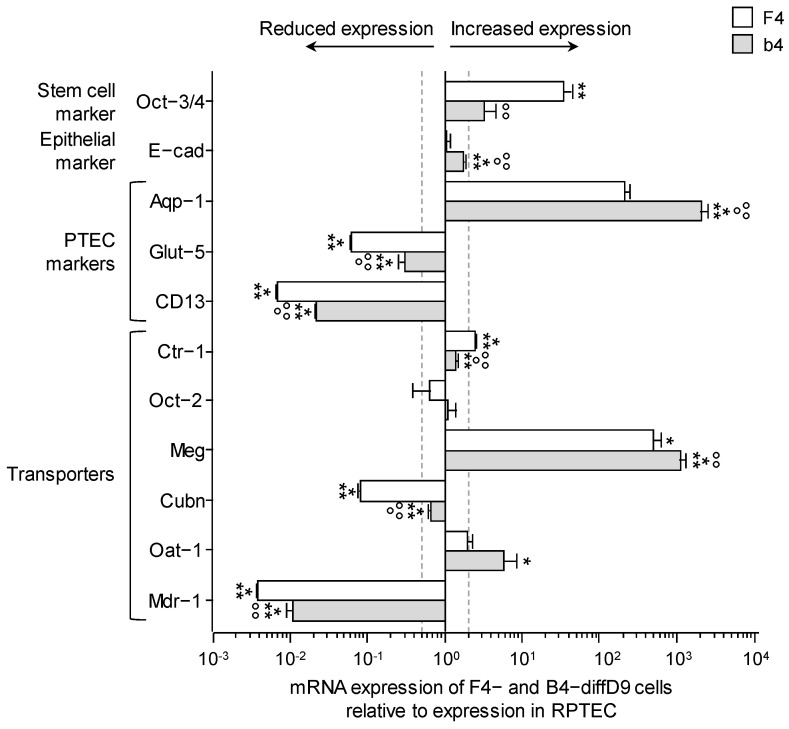
Expression changes in selected genes in F4- and b4-hiPSC differentiated into proximal tubular epithelial cell (PTEC)-like cells compared to the expression in a commercial renal proximal tubule epithelial kidney cell line (RPTEC/TERT1). mRNA expression of a stem cell marker, an epithelial marker, several PTEC markers, and transporters relative to expression in RPTEC/TERT1 were analyzed by quantitative RT-PCR. Data from at least 3 independent experiments (qPCR also included 3 technical replicates) are shown as mean ± SD. * *p* ≤ 0.05, ** *p* < 0.01 and *** *p* < 0.001 vs. RPTEC/TERT1, °° *p* < 0.01, °°° *p* < 0.001 vs. F4 (ANOVA). BSA = bovine serum albumin, DAPI = 4′,6-diamidino-2-phenylindole, diffD9 = differentiation day 9, FITC = fluorescein isothiocyanate, hiPSC = human induced pluripotent cell. Aqp-1 = aquaporin-1, CD13 = alanyl aminopeptidase, Ctr-1 = copper transporter 1, Cubn = cubilin, diffD9 = differentiation day 9, E-cad = E-cadherin, Glut-5 = fructose transporter 5, hiPSC = human induced pluripotent cells, Meg = megalin, Oct-3/4 = octamer-binding transcription factor 3/4, RPTEC = renal proximal tubule epithelial cells.

Gene expression analysis of selected genes in PTELC differentiated from F4- or b4-hiPSC showed up-, down-, and unregulated expression of genes in the differentiated cells (Figure 5). Expressed at the same level as in RPTEC/TERT1 were the transporter *Oct-2* in both PTELC lines, and *E-cadherin*, and *Oat-1* in PTELC originating from F4-hiPSC. The pluripotent stem cell marker *Oct-3/4*, one marker of the proximal tubules, *Aqp-1*, and a subunit of the albumin transporter, *megalin* were highly increased compared with expression in RPTEC/TERT1. The expression of all other genes examined was downregulated compared with the expression in RPTEC/TERT1.

### 2.4. Sensitivity of hiPSC, hiPSC Differentiating into PTELC, and hiPSC Differentiated into PTELC towards a Genotoxic Nephrotoxin and a Non-Genotoxic Nephrotoxin

**Figure 6:**
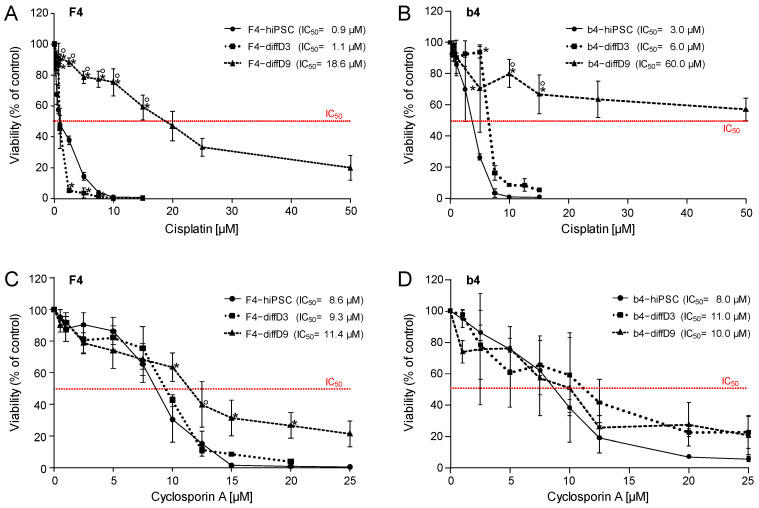
Sensitivities of hiPSC and cells on differentiation days 3 and 9 to selected nephrotoxins. Cell viability was measured with the Alamar Blue assay on F4- (**A**,**C**) and b4- (**B**,**D**) cells of differentiation days 0 (hiPSC), 3 (diffD3), and 9 (diffD9) after 24 h treatment with (**A**,**B**) cisplatin and (**C**,**D**) cyclosporin A in the indicated concentrations. The concentrations at which 50% of the cells were dead are given as IC_50_ values in the graphs. Data from at least 3 independent experiments are shown as mean ± SD. * *p* ≤ 0.05 vs. hiPSC, ° *p* ≤ 0.05 vs. diffD3 (One-way ANOVA).

hiPSC, hiPSC in the process of differentiating into PTELC on differentiation day three, and hiPSC after nine days of differentiation were treated with two different nephrotoxins. Figure 6A shows the response of the cells to the genotoxic nephrotoxin cisplatin. The highly proliferating F4-hiPSC and differentiating F4-hiPSC showed the same sensitivity towards cisplatin, with an IC_50_ of around 1 µM. F4-diffD9 cells were not as sensitive, with an almost twenty times higher IC_50_ of 18 µM. In contrast to results obtained with F4-cells, b4-hiPSC and differentiating b4-hiPSC did differ by a factor of two in their IC_50_ of cisplatin, with the diffD3 cells showing a lower sensitivity. The differentiated b4-hiPSC are also the least sensitive cells among the different states of the b4 cells. Their IC_50_ is 10 to 20 times higher than b4-hiPSC and differentiating b4 (Figure 6B). In total, the b4 cells seemed to be more resistant to cisplatin than the F4 cells, although diffD3- and diffD9-b4 had a higher proliferation rate. Regarding the non-genotoxic cyclosporin A, cells of all three differentiation states of both F4 and b4 showed the same sensitivity with IC_50_ values around 10 µM (Figure 6C,D).

### 2.5. Impact of Toxin Treatment on Marker Expression and Transporter Function

**Figure 7:**
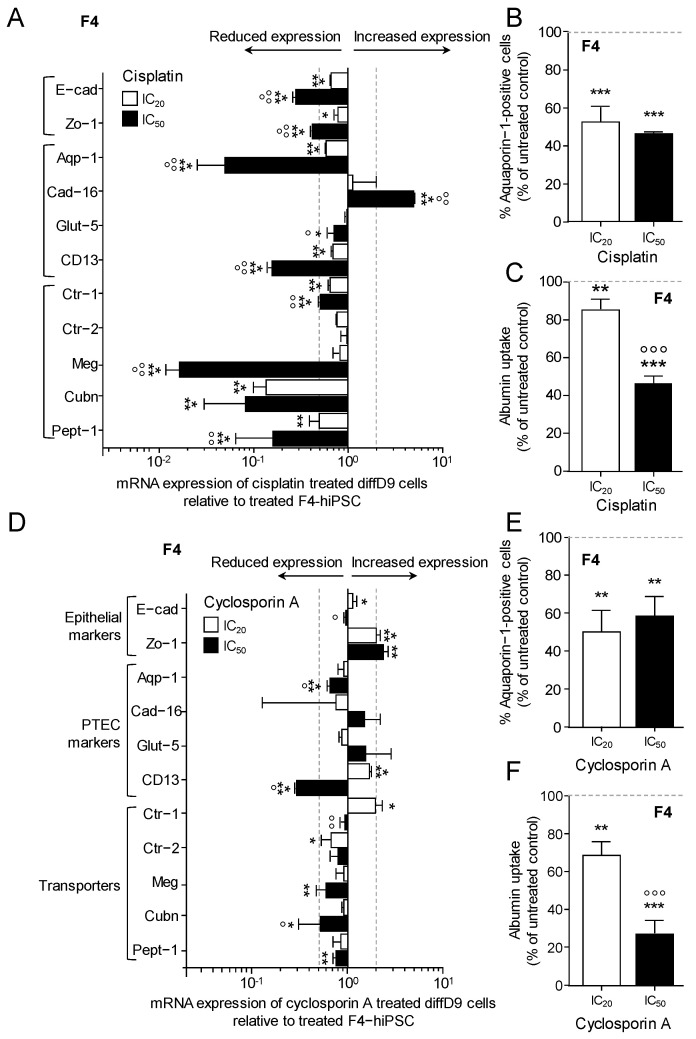
Effects of cisplatin and cyclosporin A on marker gene expression and transport function in F4-hiPSC differentiated into proximal tubular epithelial cell (PTEC)-like cells. mRNA expression of marker genes in diffD9 F4 cells after 24 h treatment with the IC_20_ and IC_50_ concentrations of cisplatin (**A**) and cyclosporin A (**D**) relative to their expression in F4-hiPSC analyzed by quantitative RT-PCR. Flow cytometry analysis of the PTEC typical protein aquaporin-1 in diffD9 F4 cells treated for 24 h with the IC_20_ and IC_50_ concentrations of cisplatin (**B**) and cyclosporin A (**E**). Flow cytometric analysis of albumin uptake into diffD9 F4 cells after 24 h treatment with the IC_20_ and IC_50_ concentrations of cisplatin (**C**) and cyclosporin A (**F**) with the help of FITC-labelled albumin. Data from at least 3 independent experiments (qPCR included 3 technical replicates) are shown as mean + SD. * *p* ≤ 0.05, ** *p* < 0.01 and *** *p* < 0.001 vs. treated F4-hiPSC, ° *p* ≤ 0.05, °° *p* < 0.01, °°° *p* < 0.001 vs. diffD9 cells treated with IC_20_ (ANOVA). Aqp-1 = aquaporin-1, Cad-16 = cadherin 16, CD13 = alanyl aminopeptidase, Ctr-1/2 = copper transporter 1/2, Cubn = cubilin, diffD9 = differentiation day 9, E-cad = E-cadherin, Glut-5 = fructose transporter 5, hiPSC = human induced pluripotent cells, Meg = megalin, Pept-1 = peptide transporter 1, Zo-1 = zonula occludens-1.

For further studies on the influence of toxins on the function of differentiated cells, we focused on F4 cells. Treatment of the diffD9 cells with the IC_20_ and IC_50_ of cisplatin resulted in a significant decrease in the expression of almost all tested genes except *Ctr-2,* as well as *megalin* and *Glut-5* at IC_20_ (Figure 7A). *Cad-16* was the only gene to show a significant increase after treatment with the IC_50_. A dose-dependent response was also observed for almost all genes. The reduced expression of the PTEC marker *Aqp-1* could also be observed at the protein level, but no dose dependency was found here. IC_20_ lowered Aqp-1 expression by 50%, just like IC_50_ (Figure 7B). Furthermore, a significant dose-dependent decrease in albumin transport was detected, possibly caused by a reduction in the expression of the responsible transporter components, *megalin* and *cubilin* (Figure 7C).

The IC_20_ and IC_50_ of cyclosporin A did not have as pronounced an effect on the expression of the selected genes as cisplatin did. Although the expression of some genes was significantly decreased or increased, only *Zo-1*, *CD13*, *Ctr-1*, and *cubilin* approached or exceeded the 0.5- or 2-fold change in mRNA levels set as biologically relevant (Figure 7D). IC_50_ caused higher changes in expression than IC_20_. Identical to cisplatin, a dose-independent reduction in the protein expression of the specific marker of proximal tubule cells, Aqp-1, was observed after cyclosporine A treatment (Figure 7E). This was reduced to 50% by cisplatin, although mRNA levels had decreased less. Even more pronounced than after cisplatin treatment, albumin transport was decreased by cyclosporin A (Figure 7F). The only slightly reduced expression of *megalin* and *cubilin* after cyclosporin A treatment may not have been the only relevant factor for this effect.

## 3. Discussion

For the first time, hiPSC, hiPSC differentiating into PTELC, and hiPSC differentiated into PTELC were studied for their sensitivity to selected nephrotoxins. These included the genotoxic nephrotoxin cisplatin and the non-genotoxic nephrotoxin cyclosporin A. Two hiPSC lines, F4 and b4, were employed here to assess whether the effects were similar. The cells at different stages of differentiation showed different sensitivity to the nephrotoxic agents tested, which may be explained in part by the nature of the agents.

The differentiation of the F4 and b4 hiPSC using the protocol published by Kandasamy et al. [13] changed the morphology of the cells to a renal cell-like morphology, which allowed conclusions about the functional competence of the cells, as they formed tubular-like structures and domes. Dome or hemicyst formation occurs by polarized epithelial cells under culture conditions performing trans-epithelial fluid transport [19]. Furthermore, the strong reduction in proliferation over the time span of the differentiation process equally underlines the distinguished degree of differentiation of the cells after 9 days.

In agreement with Kandasamy et al. [13], most of the markers analyzed in F4 and b4, such as the pluripotent stem cell markers *Oct3/4*, *Nanog*, and *Sox2*, the epithelial markers *E-cadherin* and *zonula occludens-1*, the PTEC markers *cadherin-16* and *aquaporin-1*, and five out of nine transporters, showed a comparable change in the levels of mRNA expression. The post-differentiation expression levels are also consistent with the data of overlapping markers of PTELC differentiated from hiPSC by another research group using an utterly different differentiation protocol [16]. Consistent with the mRNA data, Oct-3/4 levels were also reduced at the protein level after differentiation, as demonstrated by immunocytochemistry, as also shown by Chandrasekaran et al. [16]. At the same time, the expression of epithelial and PTEC markers increased with the correct cellular localization, except CD-13, which is consistent with the corresponding markers studied by the other two groups [13,16]. Moreover, more than 95% of F4 cells were positive for the PTEC marker, aquaporin-1, after differentiation, as quantified by flow cytometry, indicating that the differentiation process was quite efficient, which was also observed by Kandasamy et al. [13].

However, an important aspect that must be considered when differentiating cells from hiPSC and analyzing expression differences is that RT-qPCR cannot determine an absolute number of genes but only a relative number. This means that the expression of the differentiated cells is related to the expression of the undifferentiated hiPSC. Nevertheless, it is not known how much of the examined mRNA the hiPSC has already expressed under basal conditions. To reach the pluripotent state during hiPSC reprogramming, fibroblasts undergo a mesenchymal-to-epithelial transition, losing mesenchymal markers and gaining epithelial ones [20]. They also form adherens junctions, tight junctions, and polarity [21,22]. This could explain the minor changes in epithelial markers observed in the gene expression analyses, which were inconsistent with the corresponding protein expression analyses.

In addition to gene and protein expression patterns similar to those of PTEC, PTELC also expressed essential tubular transporters such as megalin and cubilin, which are responsible for the uptake of albumin, an important function of the proximal tubule and were more highly expressed after differentiation than in F4 and b4. The incorporation of albumin further demonstrates the functionality of our differentiated PTELC, similar to that of LLC-PK1, which served as a positive control. RPTEC/TERT1, on the other hand, had reduced albumin uptake activity. The lack of functional albumin uptake in RPTEC/TERT1 cells has already been reported [16,20]. Some of the other studied transporters, such as *Oct-2*, *Mdr-1*, and *Sglt-2*, decreased their mRNA expression levels compared to the hiPSC. For Oct-2, one of the transporters responsible for cisplatin uptake [23,24], we could show no significant difference at the protein level in F4, in contrast to the result of reduced mRNA expression. As cell epithelialization seems crucial to gain pluripotent characteristics [21], it can be expected that further genes characteristic for epithelial cells besides the common epithelial markers currently tested are already basally expressed by the hiPSC, for example, transporters. Reports indicate that pluripotent stem cells already express, e.g., the glucose transporter Sglt2 [25,26], which appears strongly downregulated in our differentiated cells compared to the hiPSC.

When investigating the sensitivity of cells at different stages of differentiation to cisplatin, it was found that the defense system of the cells against cisplatin must have changed considerably with the differentiation process, as the undifferentiated hiPSC of both lines were the most sensitive, followed by the differentiating cells, while the fully differentiated cells were the least sensitive. This was expected because as a DNA-damaging agent, cisplatin interferes with cell division, and the proliferation rate of the differentiated cells was much lower than that of the not-yet-differentiated cells. Surprisingly, the differentiating F4 cells were as sensitive as the F4-hiPSC, even though the former already exhibited greatly reduced proliferation. Future experiments are needed to reveal whether this comparable sensitivity in the presence of reduced proliferation may be due to the differentiating cells being more susceptible to the cytotoxic effects of cisplatin. These include mitochondrial damage, direct activation of apoptosis via the TNF family, and triggering of stress at the endoplasmic reticulum (as summarized by [21]). However, in the case of b4, the differentiating cells had not yet downregulated their proliferation as much on differentiation day 3, so similar sensitivity to cisplatin was not surprising here.

A few studies have examined the dose-response to cisplatin in stem cells and differentiated cells. The IC_50_ values of our F4- and b4-hiPSC were in the range of other stem cells, and hiPSC and hESC were equally sensitive [22,23]. A two- to three-fold increase in sensitivity between two cell lines, as we saw with b4 and F4, has already been observed by others [22]. Stem cells derived from amniotic fluid alone were less sensitive [24]. Cells differentiated from stem cells lose viability only at 10- to 100-fold higher cisplatin concentrations [25,26], as did ours. Even far less sensitive to cisplatin are renal organoids generated from stem cells or differentiated PTELC growing on beads [18,27]. We are aware of only one study that determined the sensitivity of the differentiated cells as well as the sensitivity of the original hiPSC cells, as we did. Here, the IC_50_ of the hiPSC was in the same range as in our hiPSC, whereas the neurons differentiated from them were so resistant that no IC_50_ could be determined [22]. To date, no study has measured the sensitivities of cells in the process of differentiation. Cell stages derived from b4 were overall less sensitive (3 to 6 times) to cisplatin than those derived from F4. So far, we have no explanation for this. A possibility might be different levels and activities of Mdr-1, which is a known exporter of cisplatin, and which needs to be studied in the two hiPSC lines in the future. A take-home message from these observations is the importance of analyzing several hiPSC lines when carrying out such studies due to inherent variabilities between hiPSC lines.

With respect to cyclosporin A, the cellular defense system against this substance did not appear to have changed significantly with differentiation as the cells showed concordant sensitivities, suggesting a more general cytotoxic mechanism of cyclosporin A. Effects of cyclosporin A have not been studied in detail in stem cells. Therefore, IC_50_ values for cyclosporin A from stem cells or differentiating cells are not available in the literature so far. Most evidence of cyclosporine A in association with stem cells is related to the improved survival of transplanted hiPSC-derived neural precursor cells due to administered cyclosporine A (i.e., reviewed by Antonios et al. [28]). Only hiPSC differentiated into brain-like endothelial cells were investigated with regard to their sensitivity to cyclosporin A and showed an IC_50_ value of more than 15 µM after 7-day incubation [29], which is not comparable to the value of our differentiated cells, which already exhibit a lower IC_50_ after 24 h. In monocytes, on the other hand, an IC_50_ value was determined at approximately 5 µM after 72 h [30]. In relation to non-stem cells, the literature on cyclosporin A contains very contradictory information. On the one hand, cyclosporin A induces apoptosis, leading to mitochondrial damage and EMT at concentrations between 1 and 5 µM [30,31,32]. On the other hand, it protects against apoptosis and EMT induced by other substances at similar concentrations [33,34]. Future studies will reveal which of the acute effects is causing the observed cytotoxicity in our model.

Cisplatin and cyclosporine A treatments not only reduced the viability of the cells tested here but also impaired the function of the fully differentiated cells and the expression of markers associated with differentiation. Thus, cisplatin, in particular, induced decreased mRNA expression of a number of transport proteins and reduced albumin uptake. It is known that cisplatin binds to megalin, preventing it from absorbing its ligands, which include albumin [35]. This could explain the reduced albumin uptake we saw after cisplatin treatment. Other nephrotoxins, such as aristolochic acid, are also known to reduce the expression of megalin [36]. To our knowledge, this has not yet been reported for cisplatin. However, the mechanism exerted by these toxins seems to be via the upregulation of TNF-α [36], which is also increased by cisplatin [37]. Aquaporins, on the other hand, have been documented to be reduced in expression by cisplatin exposure, which we also observed, but the mechanism is not yet known for aquaporin-1 [38,39]. Cyclosporin A is also known to inhibit various transporters, among them the Mdr-1, peptide transporter-1 (Pept-1), organic anion transporters, and sodium transporters [40,41,42]. However, no change in the expression of these transporter molecules could be detected [42,43]. Other transporters, such as sodium transporters, are downregulated by cyclosporine A treatment in vivo, but this is most likely related to a change in blood pressure [44]. Regarding the impact of cyclosporin A on the gene expression of other transporters, only indirect hints could be found; for example, in rats with kidney transplants, the expression of several transporters (among them, *glucose transporter 3*, *sodium-dependent phosphate cotransporter*, and *nucleoside transporter 2*) was lower in the animals receiving cyclosporin A compared to the non-treated animals [45]. No evidence of cyclosporin A reducing the expression of *megalin* could be found. Because the reduction in mRNA of *megalin* and *cubilin* was higher in cisplatin-treated cells than in cyclosporin A-treated cells, but albumin uptake was more impaired in the latter, the loss of expression cannot be the only cause of reduced albumin uptake.

Overall, the differentiation protocol adapted from Kandasamy et al. [13] resulted in cells that resemble proximal tubule cells not only morphologically but also in their mRNA and protein expression pattern. Of course, only part of the transcriptome was verified, but important PTEC markers could be found in the differentiated cells. Functional albumin transport was also detected. The sensitivity of the cells to cisplatin changed dramatically during differentiation, which was only partly due to the decrease in proliferation rate. In contrast, all cell stages were equally sensitive to cyclosporin A. In addition, treatment with the two toxins had an adverse effect on differentiation marker expression. Surprisingly, the expression levels of the quantified transporter genes were clearly reduced by the toxins, which severely impaired the functional albumin uptake of the differentiated cells. On the other hand, it can also be assumed that the transport capacity of the kidney will be affected by the substances in vivo. An effect of cyclosporin A on the expression of various ion channels was recently demonstrated in rats and kidney transplant patients [46]. Our results lay the foundation for investigating changes in transport function in newly formed cells after regeneration.

The results shown here represent the first step towards a model that might be of use in the future to investigate the influence of toxins on regeneration processes in the kidney. Cells in the differentiation process are at least as sensitive, if not more sensitive, to the known nephrotoxins used here as model substances, than the finally differentiated cells. This suggests a possible vulnerability of the newly forming cells in injured tubules, which may play a role in the observed loss of kidney function after AKI. This vulnerability has yet to be confirmed in vivo as well as for other substances that may be present in the kidney during AKI.

### Limitations

While PTELC exhibited the expression of several necessary transporters and features specific to renal proximal tubule cells, including functional albumin transport (which RPTEC/TERT1, for example, are incapable of), we recognize that they have limitations. To begin with, although more than 95% of our hiPSC-derived PTELC showed increased expression of the specific marker for proximal tubules such as aquaporin 1, we cannot claim at this stage that our PTELC represent a pure population. In this context, analyses of the expression of markers characteristic of neurons, endothelial cells, podocytes, cells of the loop of Henle, distal tubule cells, and cells of the collecting duct need to be performed to exclude the existence of other cell types. To strengthen the in vivo relevance of our model, a detailed molecular and functional characterization between our hiPSC-derived PTELC and primary proximal tubular cells or tissues would help. Furthermore, like most hiPSC-based cell models, our PTELC are certainly not yet fully mature compared to adult human kidney cells and may not express the entire constellation of proximal tubule-specific genes. Adjustments to the culture conditions will therefore be necessary to achieve better maturation. This might include using other small molecules, such as CHIR99021 and TTNPB, in addition to growth factors (BMPs) to push the cells further into the renal tubular lineage. Furthermore, modifications of the culture conditions, such as different extracellular matrix components, must be exploited to mimic the 3D microenvironment present in vivo. In addition, different apical and basal media have to be tested in the future to expose the cells to toxins in a matrix more similar to primary urine. We do not claim that the model presented here can adequately represent PTEC differentiation in vivo. Instead, we propose it as a potentially helpful in vitro tool for studying the effects of toxins during renal regeneration.

## 4. Materials and Methods

### 4.1. Materials

Primers and antibodies used to detect the expression of various genes and proteins are provided in Supplementary Tables S1 and S2.

### 4.2. Methods

#### 4.2.1. Cell Culture of RPTEC, F4-and b4-hiPSC, and In Vitro Differentiation Protocol

The human primary renal proximal tubular epithelial cell line (RPTEC-TERT1) was obtained from the American Type Culture Collection (ATCC, Manassas, VA, USA) and expanded as described by [47]. In brief, cells were grown into a 1:1 composition of DMEM with high glucose and Ham’s F-12 nutrient mixture supplemented with 2 mM GlutaMAX (both from Thermo Fisher Scientific, Waltham, MA, USA), 100 U/mL Pen/strep, 5 µg/mL insulin, 5 µg/mL transferrin, 5 ng/mL sodium selenite, 10 ng/mL EGF, and 36 ng/mL hydrocortisone (all from Sigma-Aldrich, St. Louis, MO, USA). These cells were cultured and used until passage 10.

The foreskin-4 (F4)-human induced pluripotent stem cell line (further referred to as hiPSC) was purchased from the WiCell Stem Cell Bank (Madison, WI, USA) at passage 29. These cells were generated from human foreskin fibroblasts through lentiviral transfection of OCT3/4, Sox2, Nanog, and Lin28 [48]. b4-hiPSC were generated from human foreskin fibroblasts by reprogramming with OCT3/4, Sox2, Klf4, and c-myc [49]. Both hiPSC lines were routinely cultured on six-well plates coated with human embryonic stem cell-qualified Matrigel for the F4 (Corning, New York, NY, USA) and Reduced Growth Factor Basement Membrane Matrix—Geltrex for the b4 (Gibco, New York, NY, USA) in mTeSR1 medium for the F4 (StemCell Technologies, Vancouver, BC, Canada) and StemMacs medium for the b4 (Miltenyi Biotec, Bergisch Gladbach, Germany), supplemented with 10 mM Y-27632 dihydrochloride (Sigma-Aldrich, St. Louis, MO, USA) to keep them in exponential growth, and passaged twice a week at a ratio of 1:6 when cultures reached a confluency of 70–90%. For differentiation into renal proximal tubular epithelial-like cells (PTELC), a modified protocol, according to Kandasamy and colleagues [13], was used as illustrated (Figure 1A). Briefly, 3000 F4 cells/cm^2^ or 750 b4 cells/cm^2^ were seeded into 12-well plates coated with growth factor-reduced Matrigel (Corning, NY, USA) or Geltrex (Miltenyi Biotec, Bergisch Gladbach, Germany), respectively, in the renal epithelial growth medium (REGM) containing various growth factors and supplements (REGM BulletKit, Lonza, Basel, Switzerland) including 0.5% fetal bovine serum and 10 mM Y-27632 dihydrochloride. After 24 h, the cells were switched to the REGM differentiation medium without Y-27632 dihydrochloride but supplemented with 10 ng/mL of bone morphogenetic protein (BMP) 2 (Sigma-Aldrich, St. Louis, MO, USA) and 2.5 ng/mL BMP7 (Thermo Fisher Scientific, Waltham, MA, USA) and cultured under these conditions for 9 days with a daily medium change to induce differentiation towards PTELC. hiPSC-derived PTELC were either directly treated with nephrotoxic substances or harvested for further experiments on day 9.

#### 4.2.2. Analysis of Cell Viability

After 24 h treatment with vehicle controls (DMSO or basal medium) or with different concentrations of the model compounds cisplatin, a genotoxic nephrotoxin (Teva, Petach Tikva, Israel), and cyclosporin A, a non-genotoxic nephrotoxin (Enzo Life Sciences, Farmingdale, NY, USA), the cell viability of undifferentiated, differentiating, and differentiated cells was examined using the Alamar Blue Assay [50], which measures the reduction in the blue and non-fluorescent resazurin dye (Sigma, Steinheim, Germany) into pink and fluorescent resorufin by cell activity. Fluorescence intensity was measured in quadruplicates (excitation: 535 nm, emission: 590 nm on Tecan infinite 200, Tecan, Männedorf, Switzerland). Relative cell viability values were normalized to the respective vehicle controls and expressed as percentages.

#### 4.2.3. Analysis of Gene Expression (RT-PCR)

Total RNA was isolated and purified using the RNeasy Mini Kit (Qiagen, Venlo, The Netherlands) and RNase-free DNase Set (Qiagen, Venlo, The Netherlands), either manually or using the automated QIAcube (Qiagen, Venlo, The Netherlands). The RNA amount was quantified using the NanoVue Plus Spectrophotometer (GE Healthcare Life Sciences, Buckinghamshire, UK), stored at −80 °C, or immediately used for cDNA synthesis. cDNA was synthesized from 2000 ng RNA using the High-Capacity RNA-to-cDNA Reverse Transcription Kit (Thermo Fisher Scientific, Waltham, CA, USA) in combination with the RiboLock Rnase Inhibitor (Thermo Fisher Scientific, Waltham, CA, USA). The quantitative real-time polymerase chain reaction (qRT-PCR) was performed using the SensiMix SYBR Hi-ROX (C) Kit (Bioline GmbH, Luckenwalde, Germany) on a CFX96 Touch Real-Time PCR Detection System (Bio-Rad Laboratories, Hercules, CA, USA), and the data were analyzed using CFX Manager software (v3.1, Bio-Rad Laboratories, Hercules, CA, USA) according to the manufacturer’s instructions. All samples were run with three technical replicates. The Cq values obtained for genes of interest were first normalized to the mean Cq values obtained for the housekeeping genes Act-β and Rpl-32 and then to the control samples and expressed as a fold of this mean value. Changes in the gene expression of ≤0.5- and ≥2-fold were considered biologically relevant. Gene-specific primer pairs used for amplification in this study (Supplementary Table S1) were designed with the help of the NCBI (https://www.ncbi.nlm.nih.gov/nuccore/). These primer sets were tested and validated for specificity in a qPCR using different cDNA concentrations.

#### 4.2.4. Immunofluorescence Analysis

To monitor the expression of prototypical proximal tubular-related proteins, cells were fixed with 4% cold formaldehyde/PBS (Merck Millipore, Billerica, MA, USA) for 15 min, washed with PBS, and permeabilized twice with 0.1% Triton X-100/PBS (Sigma-Aldrich, St. Louis, MO, USA) for 5 min at room temperature (RT). After blocking (5% BSA/PBS, 1 h, RT), incubation with primary antibodies in the dilution indicated in Table S2 was performed at 4 °C overnight in a humid chamber. After washing with PBS, a fluorescence-labeled secondary antibody (Alexa Fluor 488/550 goat polyclonal to mouse or rabbit, Abcam, Cambridge, UK) was added and incubated in the dark (1:1000; 2 h). Nuclear DNA was stained with Vectashield containing DAPI (4′,6-diamidine-2-phenylindole, Vector Laboratories, Burlingame, CA, USA). Slides were sealed with nail polish and examined on a fluorescence microscope (BX43F Upright Microscope, Olympus, Shinjuku, Tokyo, Japan). 

#### 4.2.5. Western Blot Analysis

Cell extracts were prepared by lysis of cells in Roti-Load buffer (Carl Roth GmbH, Karlsruhe, Germany) followed by heating (95 °C for 5 min). First, 25 μg of the isolated protein was separated by SDS-PAGE (10 to 12%) and transferred to a nitrocellulose membrane. The membrane was blocked at RT for 1 h (5% non-fat milk in TBS/0.1% Tween 20) and incubated overnight at 4 °C with the primary antibodies in the dilution indicated in Table S2. After washing the membrane with TBS/0.1% Tween 20, incubation with peroxidase-conjugated secondary antibodies (1:2000) was performed (2 h at RT on a shaker). Finally, the membrane was visualized using the ChemiDox™ Touch Imaging System (BioRad, Munich, Germany).

#### 4.2.6. Analysis of Cell Proliferation by Measuring EdU Incorporation

To monitor the rate of proliferation during the differentiation of hiPSC into PTEC, the incorporation of fluorescent 5-ethynyl-2′-deoxyuridine (EdU) into S-phase cells was analyzed using the EdU-Click-488 proliferation assay kit according to the manufacturer’s instructions (Baseclick GmbH, Tutzingen, Germany). Briefly, cells cultivated on coverslips were incubated with 10 µM EdU for 2 h at 37 °C. Then, a dye reaction cocktail conjugated with Alexa Fluor 488 was added (30 min, RT in the dark), followed by washing with PBS and counterstaining the nuclei with DAPI. Finally, the percentage of EdU-positive cells was determined by fluorescence microscopy (BX43F Upright Microscope, Olympus, Shinjuku, Tokyo, Japan).

#### 4.2.7. Flow Cytometry-Based Quantitative Detection of Aquaporin-1

Flow cytometric analysis was performed for quantitative detection of the PTEC marker aquaporin 1. After trypsinization, at least 1 × 10^6^ cells were pelleted by centrifugation (400× *g*, 4 min, 4 °C) and treated with 0.5% Tween20 in PBS (RT, 15 min). After centrifugation, cells were resuspended in 3% BSA in PBS (RT; 15 min), followed by the addition of the primary antibody against aquaporin-1 (1:250). After washing twice with PBS, a fluorescently labeled secondary antibody (Alexa Fluor 488, goat, polyclonal to mouse, Abcam, Cambridge, UK) was added (1:1000; 30 min). The quantification of cells expressing aquaporin-1 was performed by flow cytometric analysis (Becton Dickinson, Accuri™ C6 plus, Heidelberg, Germany).

#### 4.2.8. Albumin Uptake Assay

The ability of PTEC to reabsorb albumin as a surrogate marker of their functionality was assessed by adding fluorescence-labeled bovine serum albumin (BSA-FITC, 10 mg/mL) (Sigma Aldrich, St. Louis, MO, USA) to the culture medium. After 2 h of incubation at 37 °C, uptake was stopped with ice-cold PBS. At this point, cells were either detached with trypsin, fixed with 0.5% cold formaldehyde in PBS (15 min, RT), and analyzed by flow cytometry (Becton Dickinson, Accuri™ C6 plus, Heidelberg, Germany) or fixed with 0.5% cold formaldehyde in PBS (15 min, RT) followed by counterstaining of cell nuclei and visualization by fluorescence microscopy (BX43F Upright Microscope, Olympus, Shinjuku, Tokyo, Japan).

#### 4.2.9. Statistical Analysis

Statistical analyses were performed using GraphPad Prism version 6 (GraphPad Software, San Diego, CA, USA). Data are presented as the mean and standard deviation of three independent experiments (*n* = 3). A comparison between the different sample groups was performed using Student’s *t*-test (multiple-comparison test) or a one-way analysis of variance (ANOVA). Statistically significant differences between groups were assumed at a *p*-value ≤ 0.05.

## Data Availability

The data presented in this study are available in the article and Supplementary Materials.

## Supplementary Materials

The following supporting information can be downloaded at: https://www.mdpi.com/article/10.3390/ijms25010081/s1.

## References

[1] Tiong H.Y., Huang P., Xiong S., Li Y., Vathsala A., Zink D. (2014). Drug-induced nephrotoxicity: Clinical impact and preclinical in vitro models. Mol. Pharm..

[2] Nieskens T.T.G., Sjogren A.K. (2019). Emerging In Vitro Systems to Screen and Predict Drug-Induced Kidney Toxicity. Semin. Nephrol..

[3] Naughton C.A. (2008). Drug-induced nephrotoxicity. Am. Fam. Phys..

[4] Ameku T., Taura D., Sone M., Numata T., Nakamura M., Shiota F., Toyoda T., Matsui S., Araoka T., Yasuno T. (2016). Identification of MMP1 as a novel risk factor for intracranial aneurysms in ADPKD using iPSC models. Sci. Rep..

[5] Berger K., Moeller M.J. (2014). Mechanisms of epithelial repair and regeneration after acute kidney injury. Semin. Nephrol..

[6] Rangaswamy D., Sud K. (2018). Acute kidney injury and disease: Long-term consequences and management. Nephrology (Carlton).

[7] Goldstein S.L., Jaber B.L., Faubel S., Chawla L.S., Acute Kidney Injury Advisory Group of American Society of Nephrology (2013). AKI transition of care: A potential opportunity to detect and prevent CKD. Clin. J. Am. Soc. Nephrol..

[8] Zink D., Chuah J.K.C., Ying J.Y. (2020). Assessing Toxicity with Human Cell-Based In Vitro Methods. Trends Mol. Med..

[9] Takahashi K., Tanabe K., Ohnuki M., Narita M., Ichisaka T., Tomoda K., Yamanaka S. (2007). Induction of pluripotent stem cells from adult human fibroblasts by defined factors. Cell.

[10] Vinken M., Benfenati E., Busquet F., Castell J., Clevert D.A., de Kok T.M., Dirven H., Fritsche E., Geris L., Gozalbes R. (2021). Safer chemicals using less animals: Kick-off of the European ONTOX project. Toxicology.

[11] Chu X., Bleasby K., Evers R. (2013). Species differences in drug transporters and implications for translating preclinical findings to humans. Expert. Opin. Drug Metab. Toxicol..

[12] Zou L., Stecula A., Gupta A., Prasad B., Chien H.C., Yee S.W., Wang L., Unadkat J.D., Stahl S.H., Fenner K.S. (2018). Molecular Mechanisms for Species Differences in Organic Anion Transporter 1, OAT1: Implications for Renal Drug Toxicity. Mol. Pharmacol..

[13] Kandasamy K., Chuah J.K., Su R., Huang P., Eng K.G., Xiong S., Li Y., Chia C.S., Loo L.H., Zink D. (2015). Prediction of drug-induced nephrotoxicity and injury mechanisms with human induced pluripotent stem cell-derived cells and machine learning methods. Sci. Rep..

[14] Morizane R., Miyoshi T., Bonventre J.V. (2017). Concise Review: Kidney Generation with Human Pluripotent Stem Cells. Stem Cells.

[15] Bajaj P., Rodrigues A.D., Steppan C.M., Engle S.J., Mathialagan S., Schroeter T. (2018). Human Pluripotent Stem Cell-Derived Kidney Model for Nephrotoxicity Studies. Drug Metab. Dispos..

[16] Chandrasekaran V., Carta G., da Costa Pereira D., Gupta R., Murphy C., Feifel E., Kern G., Lechner J., Cavallo A.L., Gupta S. (2021). Generation and characterization of iPSC-derived renal proximal tubule-like cells with extended stability. Sci. Rep..

[17] Lawrence M.L., Elhendawi M., Morlock M., Liu W., Liu S., Palakkan A., Seidl L.F., Hohenstein P., Sjogren A.K., Davies J.A. (2022). Human iPSC-derived renal organoids engineered to report oxidative stress can predict drug-induced toxicity. iScience.

[18] Ngo T.T.T., Rossbach B., Sebastien I., Neubauer J.C., Kurtz A., Hariharan K. (2022). Functional differentiation and scalable production of renal proximal tubular epithelial cells from human pluripotent stem cells in a dynamic culture system. Cell Prolif..

[19] Valentich J.D., Tchao R., Leighton J. (1979). Hemicyst formation stimulated by cyclic AMP in dog kidney cell line MDCK. J. Cell Physiol..

[20] Wieser M., Stadler G., Jennings P., Streubel B., Pfaller W., Ambros P., Riedl C., Katinger H., Grillari J., Grillari-Voglauer R. (2008). hTERT alone immortalizes epithelial cells of renal proximal tubules without changing their functional characteristics. Am. J. Physiol. Renal Physiol..

[21] Yang Y., Liu H., Liu F., Dong Z. (2014). Mitochondrial dysregulation and protection in cisplatin nephrotoxicity. Arch. Toxicol..

[22] Fukusumi H., Handa Y., Shofuda T., Kanemura Y. (2019). Evaluation of the susceptibility of neurons and neural stem/progenitor cells derived from human induced pluripotent stem cells to anticancer drugs. J. Pharmacol. Sci..

[23] Peskova L., Vinarsky V., Barta T., Hampl A. (2019). Human Embryonic Stem Cells Acquire Responsiveness to TRAIL upon Exposure to Cisplatin. Stem Cells Int..

[24] Upadhyaya P., Di Serafino A., Sorino L., Ballerini P., Marchisio M., Pierdomenico L., Stuppia L., Antonucci I. (2019). Genetic and epigenetic modifications induced by chemotherapeutic drugs: Human amniotic fluid stem cells as an in-vitro model. BMC Med. Genom..

[25] Wing C., Komatsu M., Delaney S.M., Krause M., Wheeler H.E., Dolan M.E. (2017). Application of stem cell derived neuronal cells to evaluate neurotoxic chemotherapy. Stem Cell Res..

[26] Wang M., Wang J., Tsui A.Y.P., Li Z., Zhang Y., Zhao Q., Xing H., Wang X. (2021). Mechanisms of peripheral neurotoxicity associated with four chemotherapy drugs using human induced pluripotent stem cell-derived peripheral neurons. Toxicol. In Vitro.

[27] Guo H., Deng N., Dou L., Ding H., Criswell T., Atala A., Furdui C.M., Zhang Y. (2020). 3-D Human Renal Tubular Organoids Generated from Urine-Derived Stem Cells for Nephrotoxicity Screening. ACS Biomater. Sci. Eng..

[28] Antonios J.P., Farah G.J., Cleary D.R., Martin J.R., Ciacci J.D., Pham M.H. (2019). Immunosuppressive mechanisms for stem cell transplant survival in spinal cord injury. Neurosurg. Focus..

[29] Wellens S., Dehouck L., Chandrasekaran V., Singh P., Loiola R.A., Sevin E., Exner T., Jennings P., Gosselet F., Culot M. (2021). Evaluation of a human iPSC-derived BBB model for repeated dose toxicity testing with cyclosporine A as model compound. Toxicol. In Vitro.

[30] Schultze N., Wanka H., Zwicker P., Lindequist U., Haertel B. (2017). Mitochondrial functions of THP-1 monocytes following the exposure to selected natural compounds. Toxicology.

[31] de Arriba G., de Hornedo J.P., Rubio S.R., Fernandez M.C., Martinez S.B., Camarero M.M., Cid T.P. (2009). Vitamin E protects against the mitochondrial damage caused by cyclosporin A in LLC-PK1 cells. Toxicol. Appl. Pharmacol..

[32] Nagavally R.R., Sunilkumar S., Akhtar M., Trombetta L.D., Ford S.M. (2021). Chrysin Ameliorates Cyclosporine-A-Induced Renal Fibrosis by Inhibiting TGF-beta1-Induced Epithelial-Mesenchymal Transition. Int. J. Mol. Sci..

[33] Lv Z., Xie G., Cui H., Yao Z., Shao C., Yuan W., Chen B. (2022). Cyclosporin-A reduced the cytotoxicity of propranolol in HUVECs via p38 MAPK signaling. Medicine.

[34] Wang T., Li N., Jin L., Qi X., Zhang C., Hua D. (2020). The calcium pump PMCA4 prevents epithelial-mesenchymal transition by inhibiting NFATc1-ZEB1 pathway in gastric cancer. Biochim. Biophys. Acta Mol. Cell Res..

[35] Hori Y., Aoki N., Kuwahara S., Hosojima M., Kaseda R., Goto S., Iida T., De S., Kabasawa H., Kaneko R. (2017). Megalin Blockade with Cilastatin Suppresses Drug-Induced Nephrotoxicity. J. Am. Soc. Nephrol..

[36] Mahadevappa R., Nielsen R., Christensen E.I., Birn H. (2014). Megalin in acute kidney injury: Foe and friend. Am. J. Physiol. Renal Physiol..

[37] Arjumand W., Seth A., Sultana S. (2011). Rutin attenuates cisplatin induced renal inflammation and apoptosis by reducing NFkappaB, TNF-alpha and caspase-3 expression in wistar rats. Food Chem. Toxicol..

[38] Kishore B.K., Krane C.M., Di Iulio D., Menon A.G., Cacini W. (2000). Expression of renal aquaporins 1, 2, and 3 in a rat model of cisplatin-induced polyuria. Kidney Int..

[39] Afjal M.A., Goswami P., Ahmad S., Dabeer S., Akhter J., Salman M., Mangla A., Raisuddin S. (2022). Tempol (4-hydroxy tempo) protects mice from cisplatin-induced acute kidney injury via modulation of expression of aquaporins and kidney injury molecule-1. Drug Chem. Toxicol..

[40] Shitara Y., Itoh T., Sato H., Li A.P., Sugiyama Y. (2003). Inhibition of transporter-mediated hepatic uptake as a mechanism for drug-drug interaction between cerivastatin and cyclosporin A. J. Pharmacol. Exp. Ther..

[41] Nascimento C.R., Braga F., Capella L.S., Santos O.R., Lopes A.G., Capella M.A. (2005). Comparative study on the effects of cyclosporin a in renal cells in culture. Nephron Exp. Nephrol..

[42] Motohashi H., Katsura T., Saito H., Inui K. (2001). Effects of tacrolimus and cyclosporin A on peptide transporter PEPT1 in Caco-2 cells. Pharm. Res..

[43] Shitara Y., Takeuchi K., Nagamatsu Y., Wada S., Sugiyama Y., Horie T. (2012). Long-lasting inhibitory effects of cyclosporin A, but not tacrolimus, on OATP1B1- and OATP1B3-mediated uptake. Drug Metab. Pharmacokinet..

[44] Lim S.W., Ahn K.O., Sheen M.R., Jeon U.S., Kim J., Yang C.W., Kwon H.M. (2007). Downregulation of renal sodium transporters and tonicity-responsive enhancer binding protein by long-term treatment with cyclosporin A. J. Am. Soc. Nephrol..

[45] Edemir B., Reuter S., Borgulya R., Schroter R., Neugebauer U., Gabriels G., Schlatter E. (2008). Acute rejection modulates gene expression in the collecting duct. J. Am. Soc. Nephrol..

[46] Capolongo G., Damiano S., Suzumoto Y., Zacchia M., Rizzo M., Zona E., Pollastro R.M., Simeoni M., Ciarcia R., Trepiccione F. (2023). Cyclosporin-induced hypertension is associated with the up-regulation of Na^+^-K^+^-2Cl^−^ cotransporter (NKCC2). Nephrol. Dial. Transplant..

[47] Secker P.F., Schlichenmaier N., Beilmann M., Deschl U., Dietrich D.R. (2019). Functional transepithelial transport measurements to detect nephrotoxicity in vitro using the RPTEC/TERT1 cell line. Arch. Toxicol..

[48] Yu J., Vodyanik M.A., Smuga-Otto K., Antosiewicz-Bourget J., Frane J.L., Tian S., Nie J., Jonsdottir G.A., Ruotti V., Stewart R. (2007). Induced pluripotent stem cell lines derived from human somatic cells. Science.

[49] Wang Y., Adjaye J. (2011). A cyclic AMP analog, 8-Br-cAMP, enhances the induction of pluripotency in human fibroblast cells. Stem Cell Rev. Rep..

[50] O’Brien J., Wilson I., Orton T., Pognan F. (2000). Investigation of the Alamar Blue (resazurin) fluorescent dye for the assessment of mammalian cell cytotoxicity. Eur. J. Biochem..

