# Supplementary material for: Sensitivity of Human Induced Pluripotent Stem Cells and Thereof Differentiated Kidney Proximal Tubular Cells towards Selected Nephrotoxins
Source: Int J Mol Sci. 2023 Dec 20;25(1):81. doi: 10.3390/ijms25010081 (PMC10779191; doi:10.3390/ijms25010081)
Supplement: Supplementary file 1 [file ijms-25-00081-s001.zip › ijms-2735144-supplementary.pdf]

Supplementary Table S1. Primer sequences used for real-time PCR.

Gene<br>Accession #	Forward	Reverse
**ACTB**<br>NM_001101.3	GAGCACAGAGCCTCGCC	TCATCATCCATGGTGAGCTGG
**AQP1**<br>NM_001101.3	CATCCTCTCAGGCATCACCTC	CACACCATCAGCCAGGTCATTG
**CAD16**<br>NM_004062.3	AGCACGTGTGAAGTCGAAGT	ACTGAGGTTCTGGGAAGTGATG
**CD13**<br>NM_001150.2	TGGCCACTACACAGATGCAG	CTGGGACCTTTGGGAAGCAT
**CTR1**<br>NM_001859.3	TGATGCCTATGACCTTCTAC	GAATGCTGACTTGTGACTTAC
**CTR2**<br>NM_001860.3	CTGTACTGTATGAAGGCATC	AAAGTGACACAAATACCACC
**CUBN**<br>NM_001081.3	TAGCTTCGTGAAGGTGTGGG	GACTGGAAGACGGCAGTGAA
**ECAD**<br>NM_001317185.1	CAGGACCAGGACTTTGACTT	AGATACCGGGGGACACTCAT
**GLUT5**<br>NM_003039.2	GCCAAAGTGCACCCAGAATG	GTCAGCCTCCCTTCCTTCAT
**MDR1**<br>NM_000927	AGTCGGAGTATCTTCTTC	TTGAATAGCGAAACATTGA
**MEG**<br>NM_004525.2	GCCAGTGGCCAAGAATGTGA	TCCGCGTCATCTGAACAGTC
**NANOG**<br>NM_024865.3	ACCTCAGCTACAAACAGGTGAA	AAAGGCTGGGGTAGGTAGGT
**NCAD**<br>NM_001792.4	AGGCTTCTGGTGAAATCGCA	GCAGTTGCTAAACTTCACATTGAG
**OAT1**<br>NM_004790.4	AGTATGGAGGTACTCCGGGC	GCATGGAGAGGCAGAGGAAG

**OAT3** NM_004254.3	CTTTGTGCCCTTGGACTTGC	GGAAGAGGCAGCTGAAGGAG
**OCT2** NM_003058.4	GAAGCCGAAAATATGCAAAG	TGCAGGGATTTCTACTTTTG
**OCT3/4** NM_001285986.1	ACCCACACTGCAGCAGATCA	CCACACTCGGACCACATCCT
**OCTN2** NM_001308122.1	CACCATTGTGACCGAGCAAG	AGCAGGCTTCTTTCCCATCC
**PEPT1** NM_005073.3	CAAGTGCATCGGTTTTGCCA	CTCTTTAGCCCAGTCCAGCC
**PEPT2** NM_021082.3	CTGGGAGGACAAGTGGTACA	AGTCCGTTCCTCTGCATGTT
**RPL32** NM_001007074.1	GTTACGACCCATCAGCCCTTG	CATGATGCCGAGAAGGAGATGG
**SGLT2** NM_003041.3	CAGTCTCCGGCATAGCAAGG	GGCCTGGGGCTCATTCATC
**SOX2** NM_003106.3	AGGATAAGTACACGCTGCCC	TAACTGTCCATGCGCTGGTT
**ZO1** NM_003257.4	GAGAGGATTTGTCCGCTCAG	AGGCCTCAGAAATCCAGCTT

ACTB = β-actin, AQP1 = aquaporin-1, CAD16 = cadherin 16, CD13 = alanyl aminopeptidase, CTR1/2 = copper transporter 1/2, CUBN = cubilin, ECAD = E-cadherin, GLUT5 = glucose transporter 5, MDR1 = multidrug resistance protein 1, MEG = megalin, NANOG = homeobox protein, NCAD = N-cadherin, OAT1/3 = organic anion transporter 1, OCT 2 = organic cation transporter 2, OCT3/4 = octamer-binding transcription factor 3/4, OCTN2 = organic cation/carnitine transporter 2, PEPT1/2 = peptide transporter 1/2, RPL32 = , SGLT2 = sodium/glucose cotransporter 2, SOX2 = sex determining region Y-box 2, ZO1 = zonula occludens 1

Supplementary Table S2. Antibodies used.

Protein	Order number	Company	Dilution used
**AQP1**	AB9566	Abcam	1:300
**CD13**	AB108310	Abcam	1:500
**CTR1**	AB129067	Abcam	1:25
**CTR2**	PA5-53246	Thermo Scientific	1:1000
**ECAD**	610181	BD	1:500
**GAPDH**	2118S	Cell Signalling	1:4000
**MEG**	AB236244	Abcam	1:500
**OCT2**	MB59600162	Biozol	1:500
**OCT3/4**	AB183900	Abcam	1:100
**URO-10**	SC-58889	Santa Cruz biotech	1:1000
**ZO1**	61-7300	Thermo Scientific	1:1000

AQP1 = aquaporin-1, CD13 = alanyl aminopeptidase, CTR1/2 = copper transporter 1/2, ECAD = E-cadherin, GAPDH = Glyceraldehyde 3-phosphate dehydrogenase, MEG = megalin, OCT2 = organic cation transporter 2, OCT4 = octamer-binding transcription factor 4, URO-10 = urothelial glycoprotein, ZO1 = zonula occludens 1

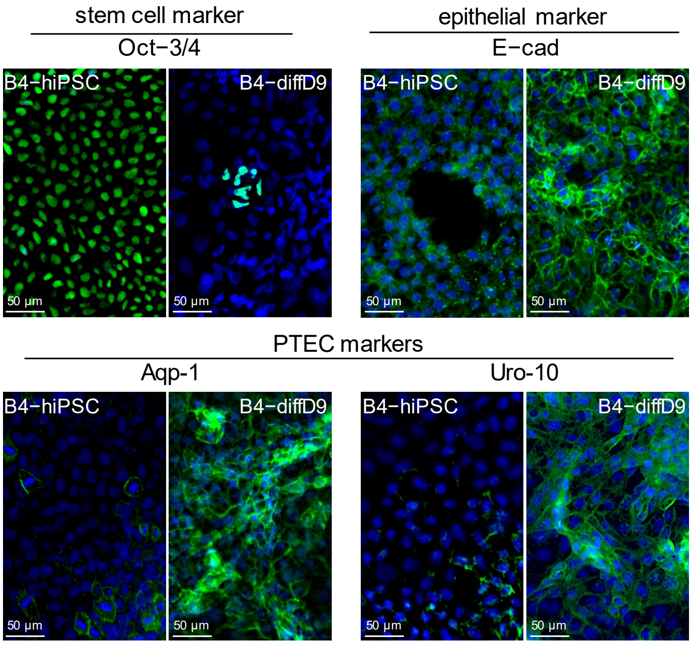

Figure S1: Expression of differentiation markers in b4-hiPSC differentiated into proximal tubular epithelial cell like cells (PTELC). Visualization of selected proteins by immunocytochemical staining on b4-hiPSC and b4 on differentiation day 9. Antibodies against the different markers are visualized with FITC-coupled secondary antibodies and nuclei are stained with DAPI. Aqp-1 = aquaporin-1, DAPI = 4′,6-diamidino-2-phenylindole, diffD = differentiation day, E-cad = E-cadherin, FITC = fluorescein isothiocyanate, hiPSC = human induced pluripotent cells, Oct-3/4 = octamer-binding transcription factor 3/4, Uro-10 = urothelial glycoprotein.
